# Enhancement of the activity of phenoxodiol by cisplatin in prostate cancer cells

**DOI:** 10.1038/sj.bjc.6604920

**Published:** 2009-02-10

**Authors:** R A C McPherson, P T Galettis, P L de Souza

**Affiliations:** 1 Cancer Pharmacology and Therapeutics Laboratory, Department of Medical Oncology, St George Hospital, Sydney, New South Wales, Australia; 2 Faculty of Medicine, University of NSW, Sydney, New South Wales, Australia

**Keywords:** isoflavones, prostate, DU145, signal transduction, Akt

## Abstract

Phenoxodiol is a novel isoflav-3-ene, currently undergoing clinical trials, that has a broad *in vitro* activity against a number of human cancer cell lines. Phenoxodiol alone inhibited DU145 and PC3 in a dose- and time-dependent manner with IC_50_ values of 8±1 and 38±9 *μ*M, respectively. The combination of phenoxodiol and cisplatin was synergistic in DU145, and additive in PC3, as assessed by the Chou–Talalay method. Carboplatin was also synergistic in combination with phenoxodiol in DU145 cells. The activity of the phenoxodiol and cisplatin combination was confirmed *in vivo* using a DU145 xenograft model in nude mice. Pharmacokinetic data from these mice suggest that the mechanism of synergy may occur through a pharmacodynamic mechanism. An intracellular cisplatin accumulation assay showed a 35% (*P*<0.05) increase in the uptake of cisplatin when it was combined in a ratio of 1 *μ*M: 5 *μ*M phenoxodiol, resulting in a 300% (*P*<0.05) increase in DNA adducts. Taken together, our results suggest that phenoxodiol has interesting properties that make combination therapy with cisplatin or carboplatin appealing.

Hormone refractory prostate cancer carries a poor prognosis, with an expected median survival of around 12 months in symptomatic patients. Recently, docetaxel in combination with either prednisone or estramustine has been shown to improve survival when compared with the combination of mitoxantrone and prednisone in phase III trials (Petrylak *et al*, 2004; Tannock *et al*, 2004). In these trials, the median survival in the control arm was only 16 months. Given that many men are unsuitable for chemotherapy, either because they are elderly or have comorbidities, there is still a pressing need to evaluate better-tolerated and more effective compounds and combinations for the treatment of this disease.

Epidemiological studies suggest an inverse relationship between isoflavone consumption and the risk of prostate cancer (Jacobsen *et al*, 1998; Strom *et al*, 1999). A significant (70%) reduction in the risk of prostate cancer was associated with the consumption of soy milk (a rich source of dietary isoflavones) in a cohort of over 12 000 Seventh Day Adventist men (Jacobsen *et al*, 1998). Although there are, as yet, no randomised trials of isoflavones in the treatment or prevention of prostate cancer, there is strong *in vitro* evidence for the activity of a variety of isoflavones on hormone-sensitive and -insensitive prostate cancer cell lines (Hempstock *et al*, 1998; Mitchell *et al*, 2000; Hedlund *et al*, 2003) and *in vivo* in rats (Risbridger *et al*, 2001). Isoflavones appear to have pleiotropic effects on prostate cancer cells, including an ability to exert hormonal influences.

Phenoxodiol is a synthetic isoflav-3-ene metabolite that is a natural intermediate (dehydroequol, 7,4′-dihydroxyisoflav-3-ene) in the metabolism of daidzein to equol (Joannou *et al*, 1995). It is cytotoxic *in vitro* (Phenoxodiol Investigators Brochure, 2000; Mor *et al*, 2006) and *in vivo* in rats (Constantinou *et al*, 2003; Mor *et al*, 2006). It may be capable of re-sensitising platinum- and taxane-resistant ovarian cancer cells *in vitro* (Kamsteeg *et al*, 2003; Sapi *et al*, 2004; Kluger *et al*, 2007) and appears to have antiangiogenic (Gamble *et al*, 2006) and anti-inflammatory properties (Widyarini *et al*, 2001). It has improved bioavailability when compared with genistein (Kelly and Husband, 2003) and low toxicity in clinical trials (Joshua *et al*, 2003; Choueiri *et al*, 2006a; de Souza *et al*, 2006).

Phenoxodiol appears to create a ‘pro-death’ environment by activating the caspase cascade through the upregulation of pro-apoptotic Bax (Alvero *et al*, 2006). It inhibits c-FLIP, activating the FAS apoptotic pathway, and causes the downregulation and cleavage of XIAP (Kamsteeg *et al*, 2003; Kluger *et al*, 2007), leading to further activation of the caspases (Straszewski-Chavez *et al*, 2004). Phenoxodiol also causes cell cycle arrest at G1 due to a loss of cdk2 activity by p53-independent induction of the cdk inhibitor p21^WAF1/CIP1^ (Aguero *et al*, 2005). Cell cycle interference occurs through the inhibition of topoisomerase II (Constantinou and Husband, 2002). The molecular target for growth inhibition by phenoxodiol is unclear, but may involve tumour-associated NADH oxidase (Yagiz *et al*, 2006).

Our aim was to determine the *in vitro* growth-inhibitory ability of phenoxodiol against prostate cancer cells, and to identify whether cisplatin could enhance these abilities, and investigate whether intracellular cisplatin uptake was altered by phenoxodiol.

## Materials and methods

### Drugs and chemicals

Phenoxodiol was supplied by Novogen Research Pty Limited (Sydney, NSW, Australia). Cisplatin and carboplatin were purchased from Sigma Chemicals (St Louis, MO, USA). Cell culture reagents and consumables were obtained from Sigma Chemicals or Invitrogen (Mulgrave, VIC, Australia). All other chemicals not otherwise specified were of the highest grade and were purchased from local suppliers.

### Cell culture

Growth of the human androgen-independent prostate cancer cell lines DU145 and PC3, as well as HepG2 (hepatoma) and 786-0 (renal), was maintained in an atmosphere of 5% CO_2_ at 37°C. DU145 cells were cultured in MEM Eagle's media, whereas PC3 cells were maintained in high-glucose RPMI-1640 media. All media were supplemented with foetal calf serum (10% v/v), 10 mM Hepes, 1.5 g l^−1^ sodium bicarbonate and penicillin–streptomycin–glutamine (1% v/v). DU145 and PC3 cells were obtained from Professor David Morris (Department of Surgery, St George Hospital, NSW, Sydney, Australia).

### Growth-inhibition experiments

Cells were plated in 96-well plates (Falcon; Becton Dickinson, Lincoln Park, NJ, USA) at 3000 cells per well in culture medium and incubated for 24 h. Drug stock solutions were diluted in culture medium and added (100 *μ*l per well) in triplicate to achieve final concentrations ranging from 0.1 to 10 *μ*M phenoxodiol, 0.001 to 100 *μ*M cisplatin and 1 to 100 *μ*M carboplatin. Drugs were investigated for their activity as single agents, and in various combinations and concentrations. After incubation for 72 h, cell viability was measured by the sulphorhodamine B assay as described earlier (Skehan *et al*, 1990). Growth inhibition was expressed as % control (media alone and no drugs) and quantitated by IC_50_ values. All experiments were repeated in triplicate; results are shown as mean±s.e.m. Schedule dependency of the phenoxodiol and cisplatin combination was also investigated by exposing cells simultaneously to both drugs for 72 h or sequentially to phenoxodiol first for 2 or 24 h followed by the addition of cisplatin for a further 48 h, or vice versa.

### Chou–Talalay analysis for synergy

Methods for assessing synergy were used as described earlier (de Souza *et al*, 1997). Briefly, Calcusyn (version 2.0), a Windows®-based computer program automating the multiple-drug-effect analysis of Chou and Talalay, based on the median-effect principle (Chou and Talalay, 1984), was used to calculate combined drug effects. The combination index (CI) equation CI=(*D*)_1_/(Dx)_1_+(*D*)_2_/(Dx)_2_ was then used to determine synergy, additivity or antagonism. Data are expressed as CI±s.e.m. Combination indices of <1, =1 and >1 represent synergy, additivity and antagonism, respectively. Mutually non-exclusive CIs are shown because these do not assume knowledge of the mechanism of action of the drugs in combination.

### Whole-cell platinum accumulation and DNA platinum-binding studies

DU145 cells were treated with cisplatin alone and in combination (1 and 10 *μ*M) with phenoxodiol at 5 *μ*M for 24 h, harvested by trypsin and then washed twice with ice-cold PBS. Samples for the whole-cell analysis of platinum accumulation were lysed with 500 *μ*l of water and analysed for total protein content by the biocinchonic acid protein assay kit (Sigma-Aldrich, Sydney, NSW, Australia). Samples for measuring platinum DNA binding were lysed in 100 mM Tris HCl, 5 mM EDTA, 0.2% SDS and 200 mM NaCl, with 100 *μ*g proteinase K per ml added immediately before use. DNA was precipitated with ice-cold isopropanol and the sample digested in 500 *μ*l TE (10 mM Tris HCl (pH 7.5) and 0.1 mM EDTA) and shaken overnight at 37°C. A total DNA measure was performed by analysis at 260/280 OD. Experiments were repeated in triplicate. Samples were diluted 1 : 5 with 0.1% nitric acid and analysed for platinum content by inductively coupled plasma mass spectrometry (ICP-MS) as described below. Platinum measures were adjusted as relevant to per mg of protein and *μ*g of DNA before comparisons between treatment groups were made.

### *In vivo* experiments

Male Nu-Nu Balb/c mice (15–20 g) were obtained from the Animal Resource Centre (Perth, WA, Australia), housed in sterile filter-topped microisolation cages and maintained on sterile water and a sterile isoflavone-free diet (Gordon's Speciality Stockfeeds, Yanderra, NSW, Australia) *ad libitum*. Mice were monitored until a bodyweight of 20–25 g was reached. Xenografts were established by the dual subcutaneous injection of 1 × 10^6^ DU145 cells in serum-free MEM and Matrigel (1 : 1) on each hind flank under inhalation anaesthesia (induced with 5% isofluorane/95% oxygen and maintained with 1–2% isofluorane). The xenografts were left to grow until approximately 20 mm^2^ in size before mice were randomised into four treatment groups (at least *n*=6 per group): vehicle only, cisplatin (1 mg kg^−1^), phenoxodiol (5 mg kg^−1^) and cisplatin plus phenoxodiol (0.5 and 2.5 mg kg^−1^, respectively). Cisplatin was prepared fresh each day, dissolved in 0.9% saline (0.1 mg ml^−1^), whereas stock phenoxodiol was prepared in 20% hydroxypropyl b-cyclodextrin (5 mg ml^−1^) solubilised at 120°C. Phenoxodiol was diluted daily in 0.9% saline. Drugs were given through the intraperitoneal route daily on days 1–5 and 8–12. Doses of cisplatin and phenoxodiol were chosen to give plasma concentrations that would approximate the concentrations used in *in vitro* experiments from literature (Litterst and Magin, 1988) and bioavailability data supplied by Novogen. Xenograft diameters were measured daily with vernier callipers and volumes calculated by the formula Volume=Length × Width^2^/2. Toxicity of the drugs was determined by inspection of mice and bodyweight analysis.

### Derivation of drug doses used for *in vivo* experiments

Doses were halved for both drugs in the cisplatin/phenoxodiol group to allow the demonstration of synergy, if present, as described earlier (de Souza *et al*, 1997). Thus, if the combination proved to be as or more effective than either agent alone at double the dose of each agent in the combination, we could conclude that the combination showed additive, or synergistic activity, respectively. The evidence for this decision is based on the Chou–Talalay equation: 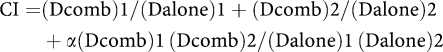 where (Dalone)1 is the dose of drug 1 alone required for a given effect (fa), (Dcomb)1 is the dose of drug 1 in the combination required for a given effect (fa), (Dalone)2 is the dose of drug 2 alone required for a given effect (fa), (Dcomb)2 is the dose of drug 2 in the combination required for a given effect (fa), CI is a measure of the degree of synergy and *α*=0 if the effects of the two drugs are mutually exclusive.

Let (Dalone)1=some concentration *p*, (Dalone)2=some concentration *q*, (Dcomb)1=0.5*p* and (Dcomb)2=0.5*q*; then CI=0.5*p*/*p*+0.5*q*/5+*α*(0.5*p*)(0.5*q*)/pq=0.5pq/pq+0.5pq/pq+0.25pq/pq. If the term *α*=0, which is likely given the different mechanisms of action of cisplatin and phenoxodiol, then CI=1, which is also the definition of additivity. The advantage of this method is that it is dose independent and does not rely on the arbitrary definition of time to recurrence/regrowth of tumours *in vivo*, for which there is no mathematical definition of synergy. As a result, only four treatment arms are required (vehicle control, drug A single-agent control, drug B single-agent control and 0.5 × drug A+0.5 × drug B in combination) for *in vivo* studies to test synergy.

### Whole-tissue platinum analysis

Total platinum in the plasma, kidney and tumours was analysed by ICP-MS as described earlier (Screnci *et al*, 1998). Tissues were prepared by nitric acid digestion overnight and for 2 h at 90°C in a closed container. Sample volume was adjusted to 10 ml with MilliQ and the platinum content analysed. Plasma (50 *μ*l) was diluted in 1.2 ml lysis buffer before analysis by ICP-MS. Calibration was against a platinum standard curve from 0.1 to 10 000 ng ml^−1^. Individual weights of tumours and kidneys were also recorded.

### Pharmacokinetics

Upon completion of the study, mice were killed at specified time points after cisplatin dosing by cervical dislocation for pharmacokinetic analysis. Cardiac puncture was performed immediately and blood was centrifuged at 2000 **g** for 5 min and the plasma supernatant stored at −80°C for cisplatin concentration measurements. At the same specified time points, the xenograft, liver and kidney tissue were also removed, washed in PBS (pH 7.6) and stored at −80°C. The data were analysed using two-way repeated measures ANOVA performed using STATVIEW version V (Abacus Concepts Inc., Berkley, CA, USA). Fisher's *post hoc* tests were performed on all significant data. Pharmacokinetic parameters were determined using non-compartmental analysis. Total platinum adjusted for tissue weight was plotted against time of sample. *C*_max_ (maximum platinum concentration) and *T*_max_ (time of maximum platinum concentration) were measured and the area under the concentration verus time curve (AUC) was calculated by the trapezoid rule (GraphPad Prism v3.00 for Windows, San Diego, CA, USA).

## Results

### Growth inhibition by phenoxodiol

**Figure 1:**
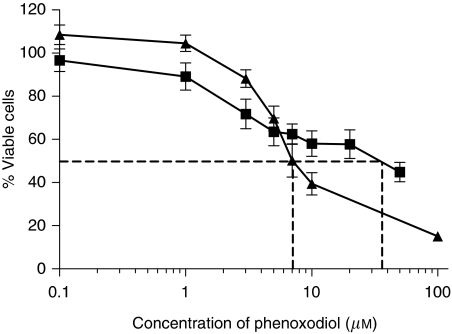
Cell viability curve following 72 h of exposure to 0.1–100 *μ*M phenoxodiol in ▪ PC3 and ▴ DU145 cells. Dashed line marks IC_50_.

Phenoxodiol was active against DU145 and PC3, with IC_50_ values of 8±1 and 38±9 *μ*M, respectively (Figure 1). Cell-growth inhibition of both cell lines was dependent on both duration of exposure and concentration of phenoxodiol.

### Synergy analysis by Chou–Talalay analysis

**Figure 2:**
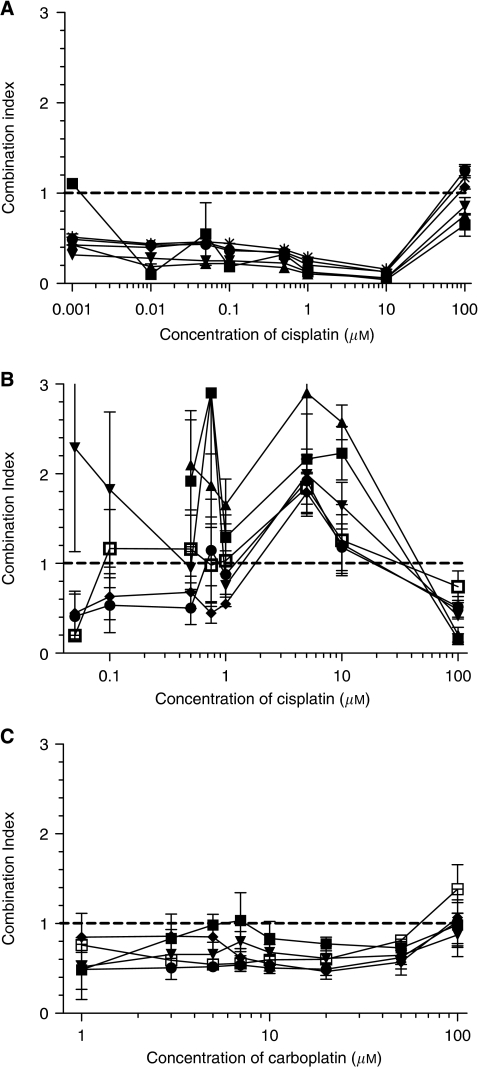
Combination indices (CI, mutually non-exclusive) for cisplatin and phenoxodiol in (**A**) DU145 and (**B**) PC3 prostate cancer cells, and (**C**) carboplatin and phenoxodiol in DU145. CI<1 denotes synergism, CI=1 additivity and CI>1 antagonism. Combination indices >3 are not shown. Phenoxodiol concentrations in (**A**), (**B**) and (**C**) are as follows: ▪ 0.1 *μ*M, ▴ 1 *μ*M, ▾ 3 *μ*M, ♦ 5 *μ*M, • 7 *μ*M, * 10 *μ*M and □ 20 *μ*M. All cells were exposed to simultaneous drug combinations for 72 h. The horizontal dashed line highlights CI=1.

Cisplatin and phenoxodiol generally showed synergistic activity against DU145 (Figure 2A) with CIs less than 1.0. Although there were some combination ratios that suggested antagonism, these occurred at the extremes of the dose–response curves. Phenoxodiol and cisplatin combined in PC3 cells to produce a mixed (synergistic, additive and antagonistic) effect that overall suggested additivity at best (Figure 2B). Synergy was also noted with carboplatin at all concentrations of phenoxodiol (0.1–20 *μ*M) against DU145 (Figure 2C). Mostly synergy interactions were found in other (HepG2 and 786-0) cell lines (data not shown).

Sequence of administration of the drugs was important. A 24-h delay between the administration of cisplatin followed by phenoxodiol significantly decreased the synergism measured (*P*<0.05), resulting in an antagonistic combination (average CI 1.8±0.5 *vs* 0.8±0.15). A 2-h delay did not affect synergism between phenoxodiol and cisplatin nor did administering phenoxodiol 24 h before cisplatin (data not shown).

### Cisplatin whole-cell accumulation and DNA binding

**Figure 3:**
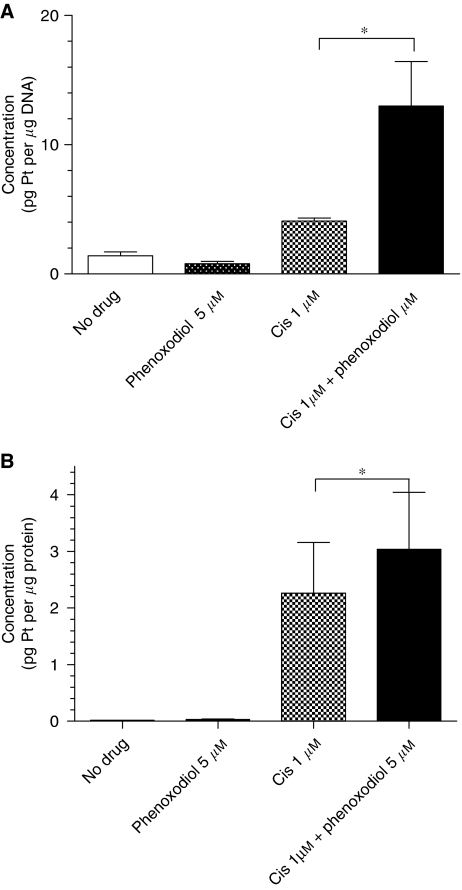
Comparison of (**A**) cisplatin DNA adducts, measured as the concentration of platinum bound to the DNA (pg platinum per *μ*g DNA) and (**B**) whole-cell platinum accumulation, recorded as the measure of platinum present in the whole-cell sample (pg platinum per *μ*g protein) following exposure to no drug (control), phenoxodiol (5 *μ*M), cisplatin (Cis) (1 *μ*M) or cisplatin (Cis) 1(*μ*M) in combination with phenoxodiol (5 *μ*M). Data shown are average level±s.e.m., ^*^P<0.05.

A greater than 300% increase (*P*<0.05) in platinum binding to DNA was measured in DU145 cells following treatment with 1 *μ*M cisplatin and 5 *μ*M phenoxodiol compared with 1 *μ*M cisplatin alone (Figure 3A). A 35% increase (*P*<0.05) in the whole-cell accumulation of platinum was measured in DU145 cells at the same concentrations (Figure 3B). No significant difference in whole-cell accumulation of platinum or DNA platinum binding was seen with 10 *μ*M cisplatin after the addition of phenoxodiol.

### Nude mice

**Figure 4:**
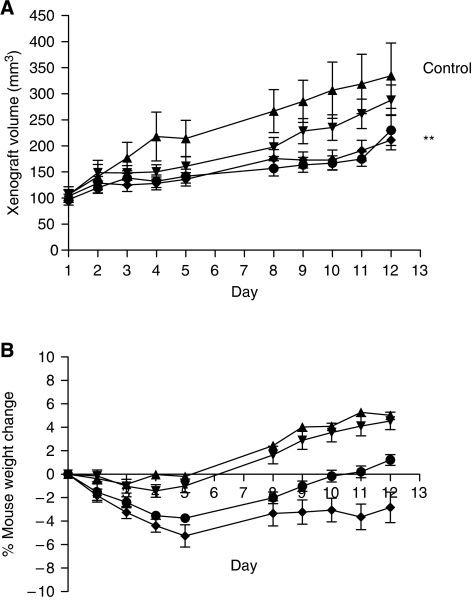
DU145 xenograft tumour volumes (mm^3^) (**A**) and nude mice % bodyweight changes (**B**) recorded over a 12-day treatment period for the four treatment groups: ▴ control, ▾ phenoxodiol alone (5 mg kg^−1^), ♦ cisplatin alone (1 mg kg^−1^) and • the combination of cisplatin (0.5 mg kg^−1^) and phenoxodiol (2.5 mg kg^−1^). ^**^A statistically significant difference (*P*<0.01) for the cisplatin arm and the combination arm compared with control; only statistically significant differences are shown (see Results).

Both cisplatin (1 mg kg^−1^) alone and the combination of phenoxodiol (2.5 mg kg^−1^) and cisplatin (0.5 mg kg^−1^) inhibited (*P*<0.01) the rate of absolute xenograft growth compared with control (Figure 4A). There was no statistically significant difference between cisplatin alone and the combination arms. Toxicity of cisplatin was monitored by recording weight loss. A statistically significant average 3.4±0.3% loss of weight was recorded for mice treated with cisplatin alone (*P*<0.01), compared with other arms (Figure 4B). Mice in the phenoxodiol and cisplatin group had less weight loss (*P*<0.01) compared with the cisplatin alone group. No mice were removed from treatment groups because of excessive weight loss (>10% bodyweight). There was no difference between mice in the control and phenoxodiol groups, in which weight gain was observed in the mice.

### Cisplatin pharmacokinetics

**Figure 5:**
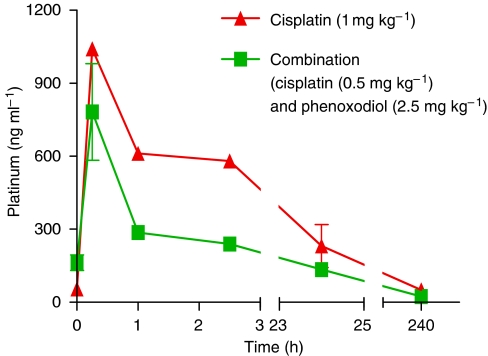
Nude mouse plasma cisplatin concentration–time curves for ⧫ cisplatin alone (1 mg kg^−1^) and • the combination of cisplatin (0.5 mg kg^−1^) and phenoxodiol (2.5 mg kg^−1^). Halving the cisplatin dose results in approximately half the AUC.

Phenoxodiol did not appear to affect cisplatin plasma pharmacokinetics. *T*_max_ was rapid and occurred at 25 min for both cisplatin alone and the cisplatin–phenoxodiol combination. The *C*_max_ for the combination of cisplatin (0.5 mg ml^−1^) with phenoxodiol (2.5 mg ml^−1^) was 782±115 ng ml^−1^ (Figure 5). This was about 75% of the *C*_max_ of cisplatin alone at twice the concentration (1 mg ml^−1^) (1041±21 ng ml^−1^). The plasma AUC of cisplatin dosed at 0.5 mg ml^−1^ was approximately half that of the 1 mg ml^–1^ group (21 950 ng ml^−1^ × h at 0.5 mg ml^−1^ in combination with phenoxodiol *vs* 40740 ng ml^−1^ × h at 1 mg ml^−1^ alone).

In kidney and tumour tissue, there was also no apparent difference in circulating levels of free platinum, with measured AUC for cisplatin at 0.5 mg ml^−1^ in combination with phenoxodiol approximately half the AUC of cisplatin 1 mg ml^−1^ alone (2914 concentration × time and 4294 concentration × time for the kidney and 812.4 concentration × time and 1861 concentration × time for the tumour respectively, data not shown).

## Discussion

The novel isoflavone, phenoxodiol, has promising *in vitro* activity against the prostate cancer cell lines DU145 and PC3, with DU145 being more sensitive than PC3. Complete inhibition of DU145 was achieved, whereas only 60% inhibition of PC3 could be achieved at the maximum solubility of phenoxodiol. Differences in sensitivity may be due to phenotypic or genetic differences between the cell lines but may also be dependent on the ability of phenoxodiol to enhance apoptosis induction. For example, PC3 cells express more Bcl-2 than DU145 (Skjoth and Issinger, 2006), and this may explain its relative resistance to treatment.

Our data show that the combination of phenoxodiol is highly synergistic with cisplatin against DU145 *in vitro* (Figure 2A). Chou–Talalay analysis of the combination shows a degree of synergy consistent with that noted when other isoflavones are combined with cisplatin (Bible and Kaufmann, 1997; Khoshyomn *et al*, 2000), suggesting that this may be a class effect. Phenoxodiol was also synergistic with carboplatin, perhaps not surprisingly, given its similar mechanism of action to cisplatin. In our hands, synergism between phenoxodiol and cisplatin is schedule dependent, with the optimal schedule appearing to be the co-administration of phenoxodiol and cisplatin, or at least phenoxodiol before cisplatin. This is consistent with the finding that cisplatin reduces cyclin D1 (Skjoth and Issinger, 2006), thereby inhibiting cell cycle progression and reducing the number of actively dividing cells available for phenoxodiol-induced apoptosis.

We speculate that phenoxodiol may interact with cisplatin in a number of ways to produce synergy in DU145 cells. DU145 cells overexpress c-FLIP short, which may in part contribute to their chemoresistance (Hyer *et al*, 2002), and the inhibition of c-FLIP by phenoxodiol has been shown in ovarian cancer cells (Kamsteeg *et al*, 2003). Phenoxodiol also induces Bax transcription (Hutson *et al*, 2003), and could therefore potentiate the sensitivity of DU145 to cisplatin (Skjoth and Issinger, 2006) as transfection of Bax in DU145 cells (naturally deficient in Bax) has been shown to significantly enhance apoptosis to a variety of chemotherapy agents including cisplatin (Honda *et al*, 2002). Modulation of sphingosine-1-phosphate by phenoxodiol (Choueiri *et al*, 2006b) may also directly alter cisplatin sensitivity. Finally, p38 induction and an intact PTEN/Akt pathway has been suggested as the mechanism for increased apoptosis in DU145 cells, relative to PC3, induced by cisplatin (Skjoth and Issinger, 2006).

The *in vivo* data are consistent with, though not strongly supportive of, our conclusions regarding the combination of phenoxodiol and cisplatin. Given that mice were treated with combination cisplatin/phenoxodiol at half-doses in the single-agent control arms, we can conclude that the combination is additive if the tumour growth curves matched, provided they were better than the negative control (vehicle only) mice, as discussed in Materials and Methods. Indeed, though the combination was better than single-agent phenoxodiol, it was no better than cisplatin alone, suggesting additivity in this xenograft experiment. The findings in our nude mice studies also support the notion that reducing the dose of both drugs and relying on their synergistic activity can not only maintain growth inhibition, but also reduce toxicity, as shown in the bodyweight curves. Further, this occurred at plasma concentrations approximately 15 times less than those found in human studies (van Hennik *et al*, 1987).

Though there was no apparent pharmacokinetic interaction between phenoxodiol and cisplatin in our *in vivo* experiment, we hypothesised that a pharmacodynamic interaction could not be ruled out. Data from DNA-binding assays seem to support this hypothesis. Phenoxodiol is not only synergistic with cisplatin in cisplatin-sensitive cell lines but also re-sensitises cisplatin- and carboplatin-resistant cell lines and tumours to the platinum agents (Mor *et al*, 2006). This also occurs with the isoflavone genistein as well as phenoxodiol's parent compound, diadzein (Gercel-Taylor *et al*, 2004). The mechanism of synergy between genistein and cisplatin is thought to be due to an increased accumulation of cisplatin within tumour cells as cisplatin accumulation of up to 83% occurs in sensitive ovarian cancer lines and 43% in resistant lines (Marveti and Andrews, 1996). Although cisplatin is not commonly used for the treatment of prostate cancer, we had earlier determined that the combination of docetaxel and phenoxodiol was only additive (unpublished), and we wished to pursue the potentially synergistic combination. Further, platins may be of renewed interest for this disease (Oh *et al*, 2007) in view of the development of satraplatin (Kelland, 2000; Petrylak, 2007; Wosikowski *et al*, 2007).

In conclusion, the combination of cisplatin or carboplatin with phenoxodiol has synergistic activity in DU145 prostate cancer cells and HepG2 hepatoma cells, and probably overall additivity in 786-0 renal cancer cells and PC3 cells. The synergy seen between cisplatin and phenoxodiol in DU145 cells may possibly be explained by an enhanced cisplatin accumulation by phenoxodiol *in vivo*. However, other mechanisms, particularly signal-transduction events, cannot be excluded. Given our promising preclinical findings, we have initiated a phase I study of the combination of either cisplatin or carboplatin together with phenoxodiol.
